# Changes in psychological distress after cancer genetic counselling: a comparison of affected and unaffected women

**DOI:** 10.1038/sj.bjc.6600030

**Published:** 2002-01-07

**Authors:** A Bish, S Sutton, C Jacobs, S Levene, A Ramirez, S Hodgson

**Affiliations:** Department of Clinical Genetics, Guy's Hospital, St Thomas's Street, London SE1 9RT, UK; Health Behaviour Unit, University College London, 2-16 Torrington Place, London WC1E 6BT, UK; ICRF Psychosocial Oncology Group, St Thomas's Hospital, Lambeth Palace Road, London SE1 7EH, UK

**Keywords:** breast and ovarian cancer genetics, affected and unaffected women, psychological distress, risk perceptions

## Abstract

This study sought to examine changes in psychological distress following cancer genetic counselling. Women attending a family cancer clinic completed questionnaires before their appointment and at 2 weeks, 6 months and 12 months after their appointment. Twenty-six women were at low risk of developing breast or ovarian cancer, 76 were at moderate risk, 46 were at high risk and 46 women had previously had breast or ovarian cancer. All groups were compared with regard to measures of anxiety, depression, general psychological distress, worry about developing breast and ovarian cancer, and perceived risk of developing breast/ovarian cancer and perceived likelihood of carrying a genetic mutation. General psychological distress did not change over the course of the study and the groups did not differ on these measures. Worry about developing breast cancer and perceptions of the likelihood of carrying a genetic mutation significantly reduced following genetic counselling. On the whole women who had already had breast/ovarian cancer showed more concerns about ovarian cancer and raised perceptions of risk in comparison with the other groups, indicating the need for sensitive counselling of such women.

*British Journal of Cancer* (2002) **86**, 43–50. DOI: 10.1038/sj/bjc/6600030 www.bjcancer.com

© 2002 The Cancer Research Campaign

## 

The population risk of developing breast cancer in the UK is one in 12 and the risk of ovarian cancer is one in 100. A small proportion (approximately 5–10%) of women who develop breast and ovarian cancers have an inherited genetic susceptibility to these cancers (Claus *et al*, 1991; Easton and Peto, 1990). To date, two breast and ovarian cancer predisposing genes have been identified – BRCA1 (Miki *et al*, 1994; Easton *et al*, 1995; Narod *et al*, 1995), and BRCA2 (Wooster *et al*, 1995). Women who have inherited a mutation in the BRCA1 or BRCA2 gene have approximately an 80% risk of developing breast cancer over their lifetime, particularly at a young age, and a 40–60% lifetime risk of ovarian cancer (Easton *et al*, 1995).

Widespread publicity about the possible genetic basis of some breast and ovarian cancers has lead to an increase in concern amongst women with a family history of these cancers. In many cases this concern may be unfounded, as the vast majority of these cancers are not due to an inherited genetic predisposition. However, increasing numbers of women are attending clinics in hospitals in the UK for genetic counselling about their family history of breast or ovarian cancer where most will want information about their future risk of developing cancer (Brain *et al*, 2000a) and about what steps they can take to minimize this risk. A further motivation for attending for genetic counselling is to undergo genetic testing.

### Psychological distress

Relevant psychological issues have been raised and discussed regarding hereditary breast cancer (Lerman and Croyle, 1994). Studies have used different assessment tools for measuring psychological distress and therefore apparent prevalence rates for distress vary (Hopwood *et al*, 1998). A variety of measures are used; some are specific to anxiety or depression (e.g. Hospital Anxiety and Depression Scale: Zigmond and Snaith (1983); Beck Depression Inventory: Beck and Steer (1987), State-Trait Anxiety Inventory: Spielberger *et al* (1983), others assess more general psychiatric distress (e.g. General Health Questionnaire: Goldberg and Williams (1988), and others are cancer specific, or can be adapted to be so (Cancer Worry Scale: Lerman *et al* (1991); Impact of Events Scale: Horowitz *et al* (1979). Comparison between samples is difficult as inclusion criteria vary.

Research in the USA has found that relatives of breast cancer patients, who are therefore at increased risk themselves, may suffer psychological distress (Kash *et al*, 1992; Wellisch *et al*, 1991). Twenty-seven per cent of the women in the study carried out by Kash *et al* (1992) were suffering psychological distress that warranted psychological counselling – although how this was assessed is not stated. Some studies in the UK and USA have found that levels of anxiety and general distress among women at risk are higher than those found in the general population (e.g. Cull *et al*, 1999; Lerman *et al*, 1994; Valdimarsdottir *et al*, 1995) although no higher than among women attending for screening.

Raised levels of distress can have detrimental effects. Women attending for genetic counselling are given information about risk and surveillance behaviours and also possible genetic testing. Women who are distressed may fail to take in this information (Hopwood *et al*, 1998; Cull *et al*, 1999) and act on it appropriately (Lerman *et al*, 1995). In addition, high anxiety may diminish women's willingness to participate in screening and surveillance (Kash *et al*, 1992; Lerman *et al*, 1993) or lead to excessive self examination (Brain *et al*, 1999).

Some of the few prospective studies that have examined how genetic counselling may influence levels of distress have found that general distress (Cull *et al*, 1999; Brain *et al*, 2000b) and cancer specific worry (Brain *et al*, 2000b; Kent *et al*, 2000) reduce following counselling, although others found no change (Watson *et al*, 1999). However, the group in the study by Watson *et al* (1999) were not split on the basis of their actual risk of developing cancer which may be likely to influence level of worry.

### The current study

The current study improves on previous research in that it includes women who have already had and been treated for breast or ovarian cancer (‘affected’ women) who make up a significant proportion of those being counselled, in addition the study classifies unaffected women in terms of their actual risk of developing breast cancer. Evaluation of the psychological effects of genetic counselling among affected women has been relatively neglected in the literature on psychological distress. The assumption seems to be that these women will be less distressed and worried in the face of being at increased risk as they have already had cancer and therefore received the ‘worst possible news’. The current study explores whether these women are in fact less distressed and worried than unaffected women. In recent years the counselling offered to affected women has increased considerably due to a changing emphasis from research to service and is now broadly similar to that offered to unaffected women. However, it does differ. Women who have already had cancer are counselled that, if they have a genetic predisposition to cancer, their chance of developing a second primary cancer would be increased. However, specific risk figures are not usually given (for women who have already had breast cancer the lifetime risk of developing ovarian cancer can be estimated based on the family history. Similarly, for women who have already had ovarian cancer the lifetime risk of developing breast cancer can be estimated based on family history). Unaffected women, on the other hand, are given an estimate of their future risk of developing cancer, with reference to epidemiological data (Claus *et al*, 1991). Current UK guidelines suggest that women estimated to have a greater than a 1 in 3 chance of developing breast cancer are at ‘high’ risk; between 1 in 6 and 1 in 4 are at ‘moderate’ risk and less than 1 in 6 are at ‘low’ risk (Eccles *et al*, 2000). It is predicted in this study that these four groups of women may differ for changes in psychological distress and worry following counselling. For example, women who are told at the first consultation that they are not at increased risk of breast cancer will show a greater reduction in specific worry about cancer than those at moderate or high risk or affected women.

The current study also assesses worry about developing ovarian cancer amongst women attending for genetic counselling. This is rarely assessed despite the fact that if women have a BRCA1 or BRCA2 mutation they are at a greatly increased lifetime risk of developing ovarian cancer. It is expected that this is not widely known by women before attending for genetic counselling. As ovarian cancer is less common than breast cancer, it is anticipated that women will be less worried about developing it than they are about developing breast cancer, but that group differences may show post-consultation once women have been informed that they may be at risk of ovarian cancer as well. For example, low risk women may be less worried than high risk women.

The main focus of this study is on psychological distress and worry, however, perceptions of risk are also relevant. There is some evidence that women attending for genetic counselling overestimate their risk of developing breast cancer before their consultation (Lerman *et al*, 1995). In contrast, Cull *et al* (2001) found that women were in fact more likely to underestimate their risk of developing ovarian cancer than to overestimate it. It is difficult to predict how risk perceptions may change following genetic counselling as this would depend on what women are told about their risk and also on how accurate their perceptions are beforehand. It is not the aim of the current study to assess accuracy of perceptions. However risk perceptions are being examined as a variable that may change following genetic counselling and which may be related to changes in worry and distress.

The aims of the current study therefore are to identify levels of distress and worry in women before they attend a genetic counselling clinic, to compare levels amongst affected and unaffected women (at differing levels of risk); to look at changes in distress and worry over time and differences among groups for any changes.

## METHOD AND MEASURES

Patients were recruited into the study at the Department of Clinical Genetics at Guy's Hospital between May 1997 and May 1999. Criteria for referral are shown in Table 1

**Table 1:**
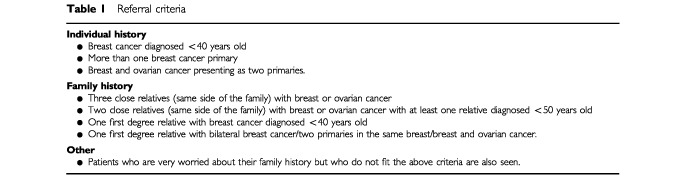
Referral criteria

. Recruitment was slower than expected and there was insufficient time to follow up all women until 12 months after their first consultation, consequently the numbers of women contacted at each time point varies. All women listed on the clinic database as due to attend their first genetic counselling appointment (*n*=577) were sent a questionnaire prior to this appointment and 412 women returned a completed questionnaire. However, 121 of the women who were sent an appointment failed to attend for this (a non-attendance rate of 21% which is in keeping with other clinics). The response rate to the questionnaire among attenders at the clinic was 86% (393 out of 456). At their consultation, all women were asked to participate in the research by completing questionnaires. Women were asked to participate regardless of whether or not they had completed the first questionnaire. In addition, some women attended the clinic unexpectedly with a relative, and so had not been sent a questionnaire previously as they had not been sent an appointment (*n*=69). As these women were also at risk, or had been affected with cancer, effort was made to include them from this stage onwards. Of the 525 women who attended the clinic during the study period 96% (505 out of 525) agreed to take part in the research and 83% of these returned a completed questionnaire in the 2 weeks following their appointment (421 out of 505). Six months later 362 women were recontacted and sent a further questionnaire. Eight-five per cent (308 out of 362) were returned. Six months after this (12 months post consultation) the final questionnaire for the study was sent to 270 women and 91% (246 out of 270) were completed. For the purposes of this study only women who had completed all four questionnaires (pre-consultation, 2 week, 6 months and 12 months post consultation) are included in the following analyses (*n*=203).

The 203 women had a mean age of 42.3 years (s.d. 12.6, range 18–79 years). Most (73%) had a partner at the time of the study and the majority (73%) had at least one child. Ninety-seven per cent of the women classified themselves as white. Most (70%) of the women currently worked outside the home.

### Genetic counselling

Women with a family history of breast/ovarian cancer who attend the family cancer clinic are either seen by a doctor (a consultant clinical geneticist or a specialist registrar in genetics) or a genetic counsellor (graduate nurses or science graduates with training in genetic counselling) for non-directive counselling. The sessions take between 45 min and 1½ h. Prior to attending the clinic women will have completed a family history sheet detailing the number of cases of cancer in their family, the type of cancer, the relationship of the person to the women, the age at diagnosis and death (if applicable). During the consultation a more detailed family history is taken. Using this information the woman's personal risk of developing breast cancer is calculated. At the time of this research the method used for risk assessment was the CASH model (Claus *et al*, 1991) which provides a risk estimate based on the number of breast cancer cases in first and second degree relatives, the age at diagnosis and the age of the woman. The basis of genetic inheritance is explained to women and the implication of genetic testing is discussed if appropriate. Options for screening and surveillance are also explained.

### Groups

Forty-six (24%) of the women had previously had breast and/or ovarian cancer (referred to in this study as ‘affected’ women): 41 had had breast cancer, one woman had had ovarian cancer and four women had had both. The mean time since diagnosis of breast cancer was 5 years (range 4 months to 32 years) and for ovarian cancer the mean time was 4 years (range 6 months to 13 years). One hundred and fifty-seven women were unaffected and had not previously had cancer. For the purposes of this study these women were classified into three risk groups (based on the current UK guidelines) using the risk estimate that was written in their hospital notes. Women had been told this figure and what it meant during their consultation. Twenty-six women (13.4%) were classified as at low risk (lifetime risk of developing breast cancer less than 1 in 6), 76 (39.2%) were classified as at moderate risk (lifetime risk of developing breast cancer between 1 in 6 and 1 in 4 inclusive) and 46 (23.7%) were classified as at high risk (lifetime risk of 1 in 3 or higher). Data were missing for nine women who are not, therefore, included in the following analyses of variance. Six of these women had not been given a breast cancer risk estimate at the clinic as their family history data was incomplete (*n*=2) or they had a family history of ovarian cancer only and their risk of developing breast cancer was therefore difficult to estimate (*n*=4). Files were missing for three women and therefore their risk could not be confirmed. The four groups of women: affected, low risk, moderate risk and high risk will be compared in the following analyses. (Forty-eight (24%) women underwent genetic testing in the course of the study: 39 affected women had blood taken to search for a BRCA1/2 mutation and nine unaffected high risk women underwent predictive testing. All had received a test result at 6 months post-consultation, although for the affected women this was a result for screening only part of the BRCA1 gene, and 37 received a result at this time stating that nothing had been found. A mutation was found in eight women and three women received a negative result on a predictive test.) With this sample size (*n*=203), power calculations showed good power to detect differences of any clinically meaningful magnitude in the variables examined in the study. For example, a sample size of 203 gives 90% power, at a 5% significance level, to detect a difference of 2.3 in breast cancer worry between two of the four groups. The power for comparisons between times will be even greater because of the within-subjects design.

### Measures

To assess psychological distress and worry three general and one specific measure were used. The Hospital Anxiety and Depression Scale (HADS; Zigmond and Snaith, 1983) gives separate measures of anxiety and depression assessed over the last week (range of scores from 0 to 21, seven items for anxiety and seven for depression). The short form of Spielberger's state-trait anxiety scale (STAI; Marteau and Bekker, 1992) was also used. This scale includes six items asking women to report their current anxiety state (range of scores from 6 to 24). The 28 item version of the General Health Questionnaire (GHQ-28; Goldberg and Williams, 1988) was used as a general measure of psychological distress (range of scores from 0 to 84). For the main analyses this continuous measure was used. However, for descriptive purposes the scale was also scored using the GHQ-scoring method and a score of 5 was taken as a cut off for case/non-case. A higher cut off of a score of 10 was also used as suggested by Hopwood *et al* (1998) for high risk women. Cronbach alpha reliabilities for the four time points in this sample for these three general measures were all greater than 0.82.

A scale designed to assess specific worry about developing cancer was used (Cancer Worry Scale; Lerman *et al*, 1991). This was adapted to create a scale assessing breast cancer worry and a separate scale to assess worry about developing ovarian cancer. (Women who had had their ovaries removed did not complete the worry about ovarian cancer scale (*n*=10). The scales each include six items scored from 1 to 4 with labelled response categories, giving a possible range of scores of 6–24. The scale includes items to assess how worry about developing cancer has affected mood and activities in the last month, and the frequency and intensity of worry. Women who had already had cancer were asked to specify their worry about developing cancer again. Cronbach alpha reliabilities for the scale at the four time points were greater than 0.82.

In addition, two measures of perceived risk were used. One assessed perceptions about breast cancer risk relative to other women of their age (scored on a 5 point Likert type scale, responses from −2 (much less likely) to 2 (much more likely)). Some research (e.g. Woloshin *et al*, 1999) suggests that such items lead to the most accurate assessment of women's perceived risk in comparison to, for example, asking women to express their perceived risk in terms of a 1 in ? chance. The second assessed perceived likelihood that they carried a mutation (scored on a 5 point Likert type scale, responses from −2 (extremely unlikely) to 2 (extremely likely)).

## RESULTS

### Missing data

Independent *t*-tests and χ^2^-tests were used to compare demographic details and baseline psychological distress and worry measures in women who did not complete questionnaires up to 12 months post-consultation with the 203 women who completed all questionnaires to check for generalizability of results. The main reason for incomplete data was lack of time in the study period to follow up all women to 1 year after their consultation, rather than it being non-response to the questionnaires. No differences were found for any of the psychological measures. However, some demographic differences were found. Women with incomplete data were younger than those with complete data (t_(418)_=2.93, *P*<0.01). They were also less likely to have a partner (χ^2^=19.7, *P*<0.001) or to have any children (χ^2^=3.9, *P*<0.05). These demographic differences are likely to be connected to one another. In addition, a higher proportion of affected women had incomplete data (χ^2^=4.6, *P*<0.05).

### Description of sample: Psychological distress and worry pre-consultation (baseline)

Prior to genetic counselling the mean score for women's levels of anxiety as measured by the HADS was 6.7 (s.d. 4.0) and for depression it was 3.0 (s.d. 2.9). Taking scores above 8 as a cut off for possible anxiety or depression disorder, 41% of the sample scored above this on the anxiety subscale and 11% did so on the depression subscale. These mean HADS scores are comparable to those found in other studies of women undergoing genetic counselling for breast/ovarian cancer risk (e.g. DudokdeWit *et al*, 1998; Kent *et al*, 2000; Lodder *et al*, 1999) and for women undergoing routine breast screening (Walker *et al*, 1994). Mean GHQ-28 scores were 21.2 (s.d. 11.5). Thirty-one per cent of women could be classified as ‘cases’ on this scale, which is comparable to proportions found by Cull *et al* (1999, 2001) and Watson *et al* (1999) amongst their samples of women undergoing genetic counselling. Using the higher threshold advocated by Hopwood *et al* (1998), only 16% of women could be classified as cases. Mean STAI scores were 11.3 (s.d. 4.2).

The pre-consultation mean score for worry about breast cancer was 12.4 (s.d. 3.4) and for worry about ovarian cancer it was 8.3 (s.d. 2.9), levels comparable to those found by Brain *et al* (1999) among women at risk of breast cancer. Thirty-four per cent of women reported that they worry often or almost all the time about developing breast cancer whereas only 7% reported these feelings about ovarian cancer. A paired *t*-test showed that, as predicted, there was significantly less worry about ovarian cancer than breast cancer pre-consultation (*t*=15.1_(188)_, *P*<0.0001). This difference persisted at 2 weeks (*t*=12.8_(188)_, *P*<0.0001), 6 months (*t*=12.1_(188)_, *P*<0.0001) and 12 months post consultation (*t*=10.0_(188)_, *P*<0.0001).

The mean score for perceptions of risk of developing breast cancer was 1.2 (s.d. 0.75) and for likelihood of carrying a mutation it was 0.8 (s.d. 0.85). Examining these variables in terms of proportions of women endorsing the given options, 47% of women felt that their risk was slightly higher than other women and a further 36% felt it was much higher. For perceived likelihood of carrying a mutation, 43% of the women felt this was fairly likely and 20% extremely likely.

### Comparison between affected and unaffected women (split by risk) for levels of, and changes in, psychological distress

Repeated measures analyses of variance (ANOVAs) were carried out to examine changes over time in anxiety, depression, general distress levels, specific worry about developing cancer and perceptions of risk and also to examine whether the groups differed on these measures. Group means (s.d.) at each of the four time points are shown in Table 2

**Table 2:**
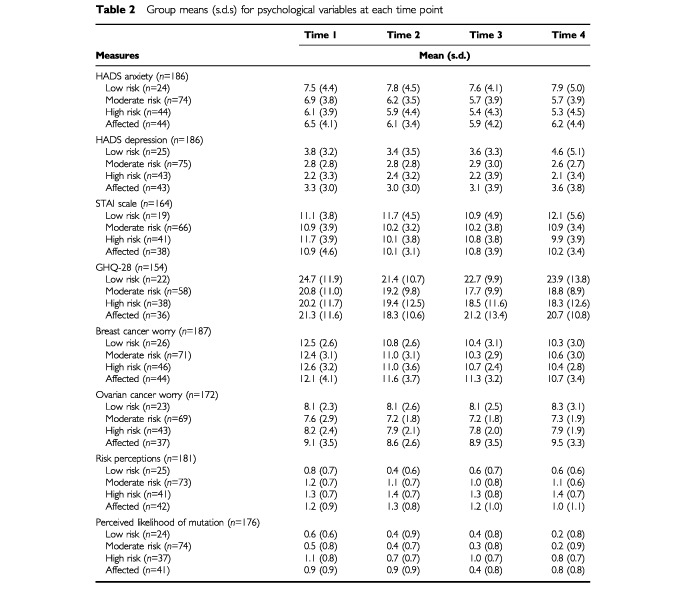
Group means (s.d.s) for psychological variables at each time point

. Results of the ANOVA are shown in Tables 3

**Table 3:**
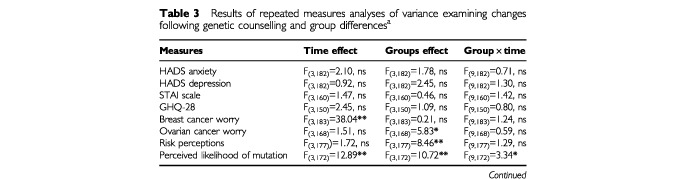
Results of repeated measures analyses of variance examining changes following genetic counselling and group differences^a^

and 4

**Table 4:**
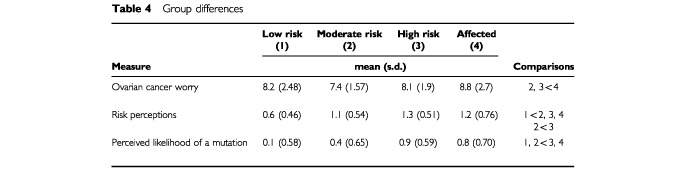
Group differences

and Figures 1

**Figure 1:**
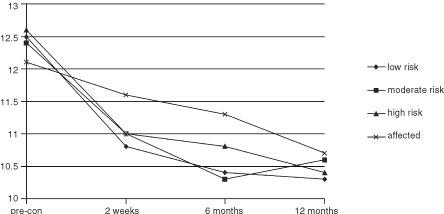
Changes in worry about breast cancer.

and 2

**Figure 2:**
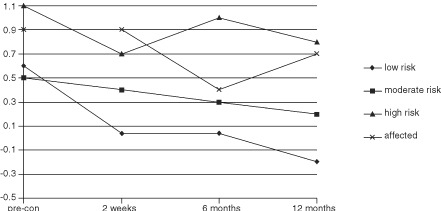
Interaction between time and group effects for likelihood of carrying a mutation.

. Supplementary analyses were also carried out to clarify the significant findings.

#### General measures (HADS, STAI, GHQ-28)

No significant changes were found in levels of general anxiety, depression, or psychological distress over the 12 month study period. In addition, the groups did not differ on these measures and there were no significant interaction effects (Table 3).

#### Worry about breast cancer

For the sample as a whole, there was a significant reduction in worry about breast cancer over the course of the study (Table 3 and Figure 1). A paired t-test was carried out in order to examine between which time points the greatest change occurred. Results indicate that the greatest reduction occurred between time 1 (pre-consultation) and time 2 (short term follow up) (*t*_(186)_=7.18, *P*<0.0001) with a further smaller reduction from time 2 to the 6 month follow up (*t*_(186)_=2.59, *P*<0.01). This was sustained until 12 months follow up with no significant further decrease (*t*_(186)_=0.82, ns). There was no group effect or interaction effect on this measure.

#### Worry about ovarian cancer

No significant changes occurred over the 12 month study period in level of worry about developing ovarian cancer. There were, however, significant group differences. A *post hoc* test (least significant difference, *P*<0.05) showed that affected women were significantly more worried than women at moderate and high risk about developing ovarian cancer. Low risk women did not differ from the other groups (Table 4).

#### Perceived risk

No significant changes occurred in levels of perceived risk over the study period. However, the groups of women did differ. A *post hoc* test revealed that low risk women perceived themselves to be less at risk than did all other groups. In addition, moderate risk women felt less at risk than did high risk women (Table 4).

#### Perceived likelihood of carrying a mutation

Over the course of the study significant changes occurred in perceptions of the likelihood of carrying a mutation (Table 3). Paired *t*-tests showed that for the sample as a whole the greatest reduction in perceived likelihood occurred between time 1 and 2 (*t*_(175)_=2.95, *P*<0.01), with no significant reduction occurring between times 2 and 3 (*t*_(175)_=1.79, ns) or 3 and 4 (*t*_(175)_=1.01, ns). In addition, groups differed on this measure. *Post hoc* tests revealed that women at low or moderate risk felt it was less likely that they carried a mutation than did women at high risk or affected women (Table 4). However, interpretation of these time and group effects needs to be qualified in light of the significant interaction between time and group found for this variable (Table 3). Individual ANOVAs were carried out for each group to confirm that significant changes occurred over time for perceived likelihood of carrying a genetic mutation. Each ANOVA was significant (low risk *F*=8.62, *P*<0.001; moderate risk *F*=4.96, *P*<0.01; high risk *F*=4.25, *P*<0.01; affected women *F*=4.13, *P*<0.01). *Post hoc* paired *t*-tests were then carried out for each group separately for changes in perceived likelihood in order to clarify the pattern of changes seen in Figure 2. Clearly, perceptions are higher among the high risk and affected women in comparison with the low and moderate risk women. The paired *t*-test results indicate that perceptions are fairly stable among the low and moderate risk women over the course of the study, whereas changes occur among high risk and affected women. Perceptions of the likelihood of carrying a gene mutation reduces for high risk women following the counselling session (*t*_(36)_=2.95, *P*<0.01), then significantly increases at 6 months post consultation (*t*_(36)_=−2.71, *P*<0.01), and significantly decreases again at 12 months post consultation (*t*_(36)_=1.87, *P*<0.05). For affected women, there is no immediate reduction in perceived likelihood (*t*_(40)_=0.00, ns), then a significant reduction at 6 months post consultation (*t*_(40)_=3.42, *P*<0.01), followed by a significant increase in perceptions at 12 months post consultation (*t*_(40)_=−2.48, *P*<0.05). For low risk and moderate risk women, other than a significant reduction following counselling for low risk women (*t*_(23)_=2.81, *P*<0.01), perceptions remain stable. For low risk women there is no change at 6 months (*t*_(23)_=0.00, ns) or 12 months (*t*_(23)_=1.81, ns). For moderate risk women there is no change immediately after counselling (*t*_(73)_=1.00, ns), at 6 months (*t*_(73)_=1.54, ns), or 12 months (*t*_(73)_=1.59, ns). Therefore, although there is enough overall change for the ANOVA to be statistically significant for these two groups (low and moderate risk women), when the pairwise *t*-tests are carried out it can be seen that the individual change between time points is small or, indeed, insignificant.

## DISCUSSION

This prospective study enabled changes in psychological distress, worry and perceptions of risk to be assessed over the course of 1 year among women at differing levels of objective risk. Prior to attending for genetic counselling, women did not have raised levels of general psychological distress, anxiety or depression in comparison to other women attending for counselling or breast screening. However, on the whole they were worried about developing breast cancer, perceived themselves to be at greater risk of breast cancer than other women their age and felt that it was likely that they carried a mutation that would increase their risk of developing cancer. Women who had already had, and been treated for, cancer were found to be particularly worried about developing ovarian cancer and perceived the likelihood of them carrying a genetic mutation to be high.

### Changes following counselling

There was no evidence found in this study that genetic counselling raises worry. Levels of worry about developing breast cancer in fact reduced following genetic counselling, regardless of what risk women had been given. The greatest reduction in worry occurred immediately after counselling, suggesting that the counselling had a positive influence rather than it simply being the passage of time that lead to a reduction in worry. Brain *et al* (2000b) also found that a reduction in worry about breast cancer was largest straight after a genetic counselling consultation. This positive effect of counselling was sustained in the long term with worry not significantly increasing, and remaining lower than at pre-consultation 12 months later. This finding is reassuring as one might expect worry to increase after counselling especially amongst the higher risk groups once women had been told about their risk. This reduction in specific worry highlights the importance of including an examination of cancer specific measures of anxiety and worry as no changes were found on any of the standard measures of general anxiety and distress. This finding is in contrast to previous research (*Cullet al*, 1999; Brain *et al*, 2000a) which found a reduction in general distress.

The study allowed for a comparison between levels of worry about developing breast and ovarian cancer. Stark differences were found, with worry being much greater about developing breast cancer. There were no changes in the level of worry about developing ovarian cancer over the course of the study which may be because level of worry was fairly low at the outset (although higher in affected women than other groups), perhaps due to the rarer nature of ovarian cancer and it receiving less media coverage. It is worth noting, however, that if a mutation is found in a woman, although ovarian cancer is less likely to develop than breast cancer, in relation to population risk it is markedly increased and perhaps worry is therefore unrealistically low.

Prior to their consultation, taking into account the rarity of BRCA1 and BRCA2 mutations, the sample of women as a whole grossly overestimated their likely chances of being a mutation carrier. However, following the genetic counselling session perceptions of the likelihood reduced most markedly. There was a significant interaction between time and group for this variable and therefore changes over time cannot be interpreted separately from group. For affected women there was a significant decrease in perceived likelihood at 6 months. This is likely to be due to 37 of these women receiving an ‘inconclusive’ result stating that two thirds of the BRCA1 gene had been searched and no mutation had been found and that the remaining third and all of BRCA2 would be searched. A study examining the interpretation of such results has been prepared for publication (Bish *et al*, 2001). This study indicates that whilst the result is in fact inconclusive it is actually interpreted by most women in a positive light as indicating that no mutation is present. At 12 months post consultation there is a significant increase in perceived likelihood again, perhaps as the time since the inconclusive result has passed and anxieties begin to increase towards pre interim result levels. For high risk women perceptions of the likelihood of carrying a genetic mutation actually increase at 6 months post consultation, which may be due to the lack of genetic testing available to most of these women at the present time and therefore their concerns over their risk are not alleviated. Low and moderate risk women show little change in perceptions, which started low and remained so following counselling. The findings concerning this variable would need to be replicated in view of the fact that only one item was used to assess perceived likelihood and therefore the measure has unestablished reliability. The pattern of change in the different risk groups is, however, interesting.

It was of interest to note that whilst worry about developing breast cancer reduced there was no change in perceptions of risk of developing this cancer, despite some women being told that their risk was low. This may be because women were reasonably realistic at the outset – those at greater risk felt more at risk. This result is in line with that found in some previous research (e.g. Leggatt *et al*, 2000). The finding may also be due to the way in which the variable was operationalized in this study where a broad base of comparison with other women was taken rather than a more specific estimate of accuracy in relation to an objective assessment. Hence women may seemingly be more ‘realistic’. The discussion of surveillance options during the consultation may have made women feel they could do something to reduce their risk, so whilst perceived risk remained the same, women were less worried. It is possible however that the measure used to assess risk was insufficiently sensitive to detect any changes since only one item assessed risk perceptions and the reliability of this measure is unknown.

### Group differences

To our knowledge the current study is the only prospective study of psychological impact of genetic counselling to include previously affected women. Affected women form a significant minority of those women being seen and the majority of those undergoing genetic testing at present. Their inclusion in psychological impact studies is therefore very important. The results of the current study have implications for practice as they show that affected women need the same level of counselling as unaffected women and that having already had cancer does not mean that the issues have been dealt with and that concerns will be fewer. For example, affected women were more worried than moderate or high risk women about developing ovarian cancer. In addition, as detailed above, the pattern of change in perceptions of likelihood show that affected women are influenced by the consultation and perceive themselves to be at, arguably, unrealistically high risk of carrying a genetic mutation.

Regarding differences between groups for risk perceptions and perceived likelihood of carrying a mutation, these were generally in line with reality – those at less risk felt at less risk. The findings are in contrast to previous research by Cull *et al* (1999) who found that a significant proportion of the women in their study continued to be inaccurate in their perceptions following counselling, even when they had been informed that their risk was low.

It is reassuring that the discussion of inherited breast and ovarian cancer did not appreciably raise anxieties in the low risk group. These women felt less at risk than other groups before counselling and afterwards. However, these women were equally as worried about developing breast or ovarian cancer as the other groups, and although their worry about breast cancer reduced it did not reduce any more than in the other groups. These low risk women may therefore constitute a group of ‘worried well’. It can be argued that their management would be more appropriate at the primary care level.

### Limitations of the study

One limitation of the current study is that it was based in only one clinic therefore the sample is fairly small, which may affect the generalisability of the results. The findings therefore need to be replicated in other clinics with different counsellors. In addition, it was not possible to follow up every woman who attended the clinic and began to participate in the research for the full 12 months. The finding that those with incomplete data were younger is in line with that found by Brain *et al* (2000b). It could be argued that the prospect of being at risk of developing cancer at a young age is too distressing for the younger women for whom it is less common among their peers. For example, Codori *et al* (1997) found that highly anxious individuals avoid risk information. However, as there were no psychological differences between groups this is unlikely to be the case. There is no evidence that the most anxious women dropped out of the study. In fact, the women participating in the research are representative of attenders at the clinics as the response rates to the questionnaires are good (over 83% for each questionnaire). A higher proportion of women in the affected group did not have complete data. The affected women included in the analyses in this study were those who had been sent an appointment, whereas many affected women attend the clinic with an unaffected relative and are counselled at this stage and may undergo genetic testing. Results from data collected from such women are included in the study by (Bish *et al*, submitted).

Past psychiatric history or use of psychotropic medication was not assessed. There is, however, no reason to suppose that these factors would systematically vary by group.

### Conclusion

In conclusion, then, specific worry about developing breast cancer was reduced for all groups following genetic counselling, although perceptions of risk did not change. Counselling had no impact on general levels of distress, which were not, however, appreciably raised before counselling. On the whole, women who have already had cancer showed raised levels of concern about developing ovarian cancer and felt more at risk of developing breast cancer and of having a mutation, in comparison with other groups. This indicates the need for sensitive counselling of such women.
